# Programmable High‐Speed and Hyper‐Efficiency DNA Signal Magnifier

**DOI:** 10.1002/advs.202104084

**Published:** 2021-12-16

**Authors:** Xiao‐Long Zhang, Yang Yin, Shu‐Min Du, Ling‐Qi Kong, Zhe‐Han Yang, Yuan‐Yuan Chang, Ya‐Qin Chai, Ruo Yuan

**Affiliations:** ^1^ Key Laboratory of Luminescence Analysis and Molecular Sensing (Southwest University) Ministry of Education College of Chemistry and Chemical Engineering Southwest University Chongqing 400715 P. R. China

**Keywords:** biosensing platform construction, cancer cell biomarker assay, dual‐catalyst hairpin assembly, high‐efficiency DNA signal magnifier

## Abstract

Herein, a programmable dual‐catalyst hairpin assembly (DCHA) for realizing the synchronous recycle of two catalysts is developed, displaying high reaction rate and outstanding conversion efficiency beyond traditional nucleic acid signal amplifications (NASA). Once catalyst I interacts with the catalyst II, the DCHA can be triggered to realize the simultaneous recycle of catalysts I and II to keep the highly concentrated intermediate product duplex I‐II instead of the steadily decreased one in typical NASA, which can accomplish in about only 16 min and achieves the outstanding conversion efficiency up to 4.54 × 10^8^, easily conquering the main predicaments of NASA: time‐consuming and low‐efficiency. As a proof of the concept, the proposed DCHA as a high‐speed and hyper‐efficiency DNA signal magnifier is successfully applied in the rapid and ultrasensitive detection of miRNA‐21 in cancer cell lysates, which exploits the new generation of universal strategy for the applications in biosensing assay, clinic diagnose, and DNA nanobiotechnology.

## Graphical abstract

Unlike the conventional catalytic hairpin assembly, the programmable dual‐catalyst hairpin assembly carved out achieves the coinstantaneous regeneration of double reactants (catalyst I and II) by introducing a well‐designed DNA as motivator, possessing spectacular reaction rate, and incredible conversion efficiency.

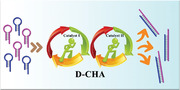

## 1 Introduction

Isothermal exponential amplification techniques, such as nuclease‐assisted strand displacement amplification,^[^ ^1^, ^2^, ^3^ ^]^ rolling circle amplification,^[^ ^4^, ^5^, ^6^ ^]^ loop‐mediated isothermal amplification (LAMP),^[^ ^7^, ^8^, ^9^, ^10^ ^]^ have been developed as alternatives to polymerase chain reaction^[^ ^11^, ^12^ ^]^ for the elimination of the large and expensive thermal cyclers, achieving comparable amplification yields. However, these methods are also suffered from complexity, time‐consumption, high‐cost, and lower efficiency further leading to the disability in clinical analysis.^[^ ^9^, ^13^ ^]^ Alternatively, enzyme‐free target recycling amplification (EFTRA) strategies,^[^ ^14^, ^15^, ^16^ ^]^ like hybridization chain reaction^[^ ^17^, ^18^ ^]^ and catalytic hairpin assembly (CHA),^[^ ^19^, ^20^, ^21^, ^22^ ^]^ can achieve the construction of autocatalytic circuits for exponential signal amplification which has advantage of operating with less interference from environmental conditions, high specificity, low cost, and excellent simplicity. Nevertheless, these methods also faced the challenge of low conversion efficiency and long reaction time leading to the nonspecific background leakage and limited amplification performance.^[^ ^23^, ^24^ ^]^ Recently, although some methods based on self‐replication could partly accelerate the signal amplification rate and reduce the reaction time to some extent,^[^ ^20^, ^25^ ^]^ the lower utilization of raw material and less effective reaction between various of reactants and resultants still cause more side reactions and low conversion efficiency. These remained issues make it a critical bottleneck to power the EFTRA with high rate and hyper efficiency.^[^ ^26^, ^27^, ^28^, ^29^ ^]^

**Figure 1:**
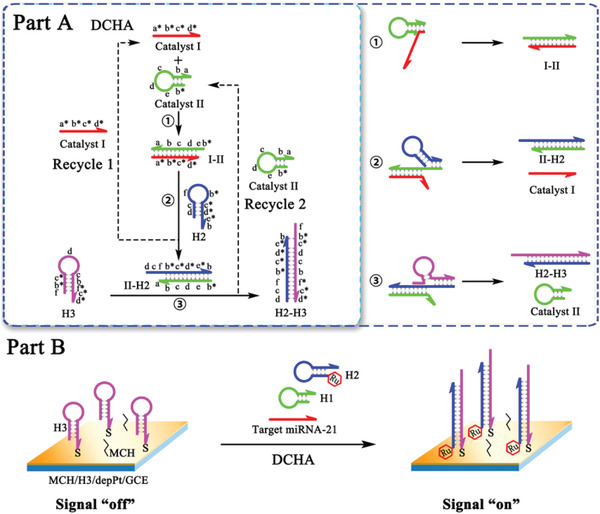
Rational design of DCHA. (Part A) Schematic illustration of the dual‐catalyst hairpin assembly (DCHA). (Part B) Illustration of the ECL biosensor construction based on DCHA for miRNA‐21 detection.

To address the problem above, herein, an original dual‐catalyst hairpin assembly (DCHA) system was developed by adding a motivator DNA strand in the traditional CHA system to achieve the synchronous recycle of two key reactants as catalysts, accompanying with constantly produced intermediate product which could endow DCHA with dramatically increased reaction speed and high conversion efficiency. As the mechanism of DCHA shown in **Figure** **1**, Part A, with the involvement of the first catalyst I (miRNA‐21) as an initiator, the catalyst II (hairpin H1) could be opened to form the duplex H1‐miRNA‐21, exposing the prelocked toehold domain of H1. Then, the H2 could displace the miRNA‐21 from the duplex H1‐miRNA‐21 to form duplex H1‐H2, achieving the recycle of catalyst I (miRNA‐21), which could also be realized in traditional CHA. Meaningfully, with the introduction of H3 as a motivator, the H1 could be displaced from the duplex H1‐H2 accompanying the formation of the duplex H2‐H3 to realize the recycle of catalyst II (H1), which could not be recycled in traditional CHA. Thus, the simultaneous recycle of catalyst I and catalyst II could keep relatively highly concentrated intermediate product duplex H1‐miRNA‐21 in DCHA and result in the strongly dynamically and thermodynamically favorable property, which could further address these obstacles in traditional methods and obtain striking reaction velocity and excellent amplification efficiency.

As a proof of concept, when target miRNA‐21 is used as the catalyst I, H2 is labeled with electroactive material ferrocene (Fc) or RuBpy (Ru), and H3 is immobilized on the gold or platinum modified electrode surface by the Au—S bond or Pt—S bond, the DCHA as a DNA signal magnifier could be applied into the construction of ultrasensitive electrochemical and electrochemiluminescent biosensor for trace detection of miRNA from cancer cells (Part B). The novel nucleic acid amplification method, we carved out showed fast DNA hybridization kinetics and hyper conversion efficiency, which gives impetus to design new one for its great potential in the biosensor construction, biological research, nanobiotechnology, and device integration.

## 2 Results and Discussion

Since the CHA is one of the most typical EFTRA with fine performance, it was chosen as a comparison group to verify the extraordinary reaction rate and outstanding conversion efficiency of the DCHA we proposed. Based on the polyacrylamide gel electrophoresis (PAGE), it was first verified that the nucleic acid hybridizations in DCHA (Figure 1 ①, ②, and ③) and CHA (Figure 1 ① and ②) could successfully proceed (Figure S1, Supporting Information). In the presence of initiator miRNA‐21, the resultants duplex H2′‐H3′ (Figure S1, lane 7, Supporting Information) and duplex H1‐H2′ (Figure S1, lane 6, Supporting Information) can be formed as expected in DCHA and CHA, respectively. We then adopted the fluorescence analysis to investigate the dynamic and thermodynamic performances of DCHA and CHA, respectively.

### 2.1 Real‐Time Fluorescence Monitor of DNA‐21 and H1 in DCHA and CHA

**Figure 2:**
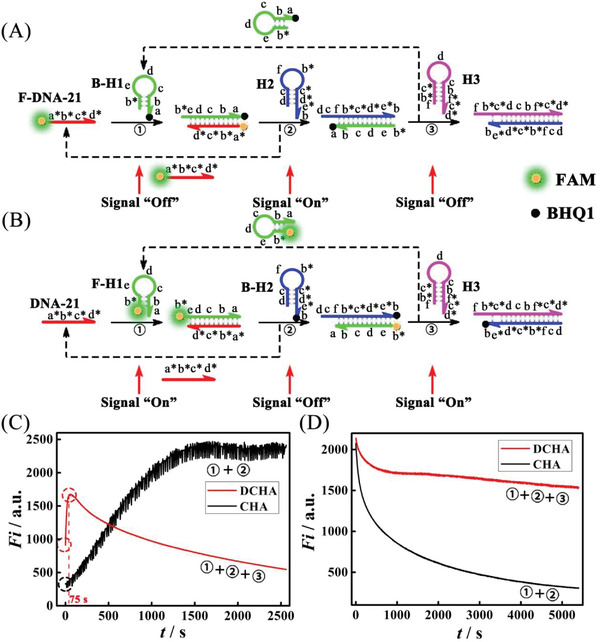
Verification of the recycle of catalysts I and II. Schematic illustration of the fluorescence signal variation with: A) F‐DNA‐21, B‐H1, H2, and H3 and B) DNA‐21, F‐H1, B‐H2, and H3. Comparison of the real‐time monitoring for fluorescence signal of C) F‐DNA‐21 and D) F‐H1 in DCHA and CHA, respectively (the initial concentration of DNA strands were all 1 × 10^−6^ m; Excitation (EX) 492 nm, Emission (EM) 528 nm, EX slit 5 nm, EM slit 10 nm, Voltage 700 V).

Primarily, the real‐time fluorescence was employed to verify if miRNA‐21 and H1 could be recycled in DCHA and CHA under optimal reaction temperature (Figure S2, Supporting Information). As shown in **Figure** **2**A, in CHA, the fluorophore FAM labeled F‐DNA‐21 (the sequence of DNA‐21 is same as miRNA‐21 except that the uracil was changed by thymine) rapidly hybridized with the quencher BHQ1 labeled B‐H1 to form amounts of the intermediate product F‐DNA‐21‐B‐H1 which could be regarded as H1‐miRNA‐21, the fluorescence of FAM thus was quenched by the closed BHQ1, resulting in a low initial fluorescence (0 s). Subsequently, the H2 hybridized with the F‐DNA‐21‐B‐H1 to release the F‐DNA‐21, the fluorescence increased gradually with the formation of duplex B‐H1‐H2 since the B‐H1 could not be regenerated. After 1500 s, the fluorescence reached a plateau, verifying that amounts of F‐DNA‐21 were recycled (Figure 2C, black curve). As a contrast, in DCHA (Figure 2C, red curve), the low initial fluorescence verified that amounts of intermediate product F‐DNA‐21‐B‐H1 could also be produced quickly by the hybridization between F‐DNA‐21 and B‐H1. Next, the fluorescence increased quite more rapidly compared with the process in CHA and reached to its zenith (75 s), verifying that the F‐DNA‐21 was displaced much faster by the H2 from F‐DNA‐21‐B‐H1 due to the introduction of H3 as an activator, demonstrating that the of F‐DNA‐21 was successfully be recycled. Moreover, the fluorescence decreased continually, which might be ascribed to that the B‐H1 could be also recycled and hybridized with the recycled F‐DNA‐21 again. From the results, it can be concluded that the DNA‐21 in DCHA or CHA could all be recycled.

In order to verify the recycle of H1 in DCHA, H1 was labeled with fluorophore FAM (F‐H1) and H2 was labeled with quencher BHQ1 (B‐H2), in which the decrease of fluorescence was monitored with the formation of duplex F‐H1‐B‐H2 in DCHA or CHA (Figure 2B). As the results shown in Figure 2D, with the time went by the fluorescence of F‐H1 labeled FAM in CHA (black curve) decreased continuously from a high value (2100 a.u.) to a low value (300 a.u.), indicating that the B‐H2 hybridized with F‐H1 to form the duplex F‐H1‐B‐H2 in the presence of DNA‐21 and the content of dissociate F‐H1 decreased continually. However, in DCHA, the fluorescence intensity (red curve) just decreased slightly from 2150 to 1750 a.u. before 1000 s and then nearly maintained a plateau value after 1000 s, which demonstrated the F‐H1 was constantly recycled in the reaction process of DCHA. The results illustrated that the H1 could also be well recycled in DCHA, while this goal couldn't be realized in CHA. Additionally, the concentration change of the intermediate product F‐DNA‐21‐B‐H1 with time were also calculated, as the results shown in Figure S3 (Supporting Information), it was verified that the intermediate product in DCHA could be indeed maintained with high concentration, while the one in traditional CHA decreased constantly.

### 2.2 Kinetic Performance of DCHA

**Figure 3:**
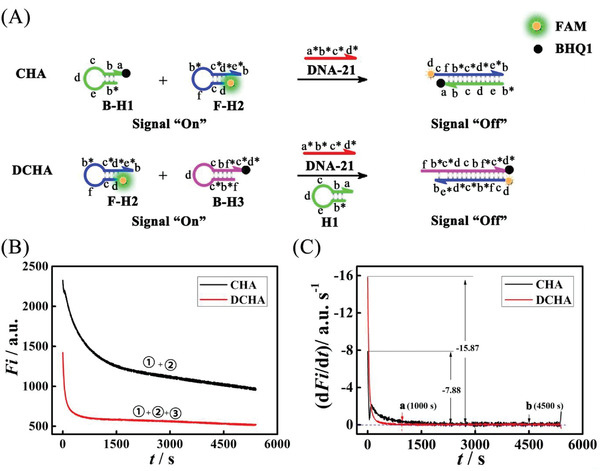
Dynamical reaction of DCHA and CHA. A) Schematic illustration of the fluorescence signal variation in CHA and DCHA. Comparison of B) real‐time monitoring of fluorescence intensity and C) the reaction rate of CHA (black line) and DCHA (red line) in response to 1 × 10^−9^ m target sequence. (The concentration of H1, H2, and H3 were all 1 × 10^−6^ m; Excitation (EX) 492 nm, Emission (EM) 528 nm, EX slit 5 nm, EM slit 10 nm, Voltage 700 V).

To quantitatively characterize the reaction rate and the conversion efficiency of DCHA and CHA, we labeled the 5′ of H2 with fluorophore FAM and labeled the 3′ of H3 and the 3′ of H1 with quencher BHQ1 in DCHA and CHA, respectively. And the DNA‐21, the sequence of which is the same with miRNA‐21 except that the uracil was changed by thymine, was used to instead miRNA‐21 as the target. This way, a fluorescence intensity decrease was measured as either the duplex H2‐H3 in DCHA or the duplex H1‐H2 in CHA was formed (**Figure** **3**A). As shown in Figure 3B, compared with the CHA (black line), the fluorescence intensity in DCHA (red line) decreased quickly to a pretty low balanced value, suggesting the quite fast reaction rate of DCHA. After deriving the fluorescence intensity with the time, the results in Figure 3C displayed that initial reaction rate of DCHA in fluorescence is obviously faster than that of CHA. While the reaction rate of DCHA and CHA approached 0, the DCHA and CHA reached a balance with the corresponding reaction time of 1000 s (point a) and 4500 s (point b), respectively. According to the ratio between the concentration of resultant and the reaction time, the calculated average reaction rate of DCHA (8.41 ×  10^−10^ m s^−1^) is about five times more than that of CHA (1.47 ×  10^−10^ m s^−1^) (the concentration of duplex H1‐H2 and duplex H2‐H3 was calculated based on Figure S5, Supporting Information). Additionally, the obtained rate constant *k* of DCHA (*k*_DCHA_ = 3.85  ×  10^3^****** m ^−1^ s^−1^) was more than twice the value of CHA (*k*_CHA_ = 1.46  ×  10^3^ m ^−1^ s^−1^) (Figure S7, Supporting Information). These results of kinetic parameters demonstrated the DCHA achieved a lot of strides in the dramatically improved reaction rate of DCHA than that of CHA, which was ascribed to that the DNA‐21 and H1 could be simultaneously recycled in DCHA, while in CHA, only the DNA‐21 could be recycled (Figure 2).

### 2.3 Thermodynamic Performance of DCHA

**Figure 4:**
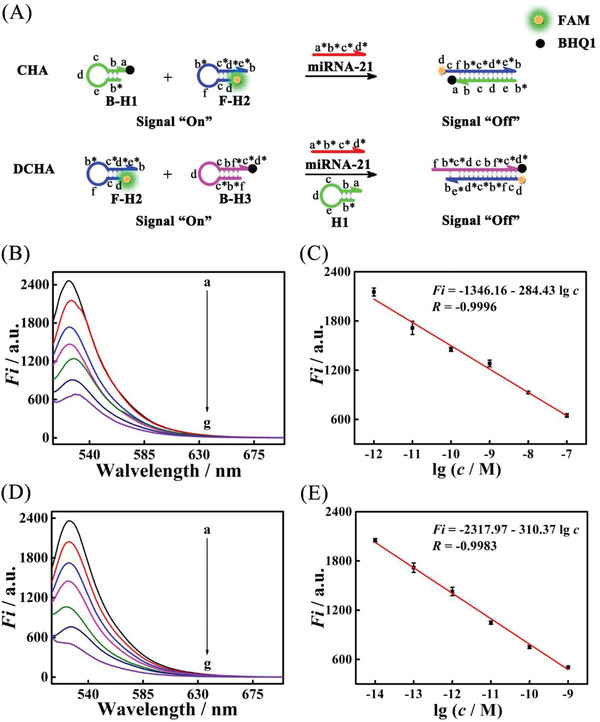
Evaluation of DCHA for miRNA sensitive response. A) Schematic illustration of the fluorescence signal variation in CHA and DCHA in the presence of miRNA‐21. B) Fluorescence responses of the CHA to different concentrations of the target miRNA‐21: a) 0, b) 1 × 10^−12^ m, c) 10 × 10^−12^ m, d) 100 × 10^−12^ m, e) 1 × 10^−9^ m, f) 10 × 10^−9^ m, g) 100 × 10^−9^ m and C) corresponding calibration plot for the fluorescence intensity versus l g *c*, *n* = 3. Error bar, mean ± SD. D) Fluorescence responses of the DCHA to different concentrations of the target miRNA‐21: a) 0, b) 10 × 10^−15^ m, c) 100 × 10^−15^ m, d) 1 × 10^−12^ m, e) 10 × 10^−12^ m, f) 100 × 10^−12^ m, g) 1 × 10^−9^ m, and E) corresponding calibration plot for the fluorescence intensity versus l g *c*, *n* = 3. Error bar, mean ± SD.

Next, to obtain the accurate conversion efficiency of DCHA and CHA, respectively, based on the fluorescence monitoring (**Figure** **4**A), we first carried out the relationship of the fluorescence response and the concentration of the quantified initiator (miRNA) in CHA (Figure 4B,C) and DCHA (Figure 4D,E), respectively. As shown in Figure 4, the fluorescence intensity in CHA and DCHA all decreased with the increase of miRNA concentration and displayed a fine linear relationship with the logarithm concentration, and the corresponding regression equation were *Fi* = −1346.16–284.43 lg *c* (from 0 to 100 × 10^−9^ m, limit of detection (LOD) = 0.36 × 10^−12^ m (Table S3, Supporting Information), correlation‐coefficient value (*R*) = −0.9996) and *Fi* = −2317.97–310.37 l g *c* (from 0 to 1 × 10^−9^ m, LOD = 6.21 × 10^−15^ m (Table S3, Supporting Information), *R* = −0.9983) separately (*Fi* and *c* represented the fluorescence intensity and miRNA concentration, separately; unit of *c* was M). And the background leakage of DCHA and CHA were also studied, as the results displayed in Figure S4 (Supporting Information), the fluorescence intensity of DCHA just changed slightly when compared with that of CHA with the time increased, suggesting that the acceptable background leakage of DCHA almost plays negligible effect on the performance enhancing.

**Figure 5:**
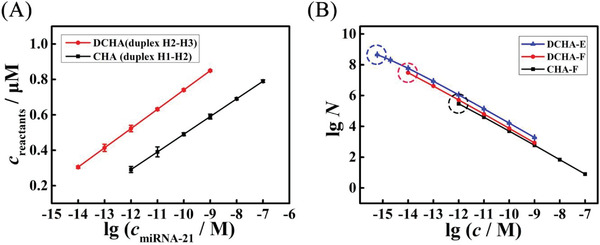
Performance of DCHA. Comparison of A) the concentration of output resultants in DCHA (duplex H2‐H3) and CHA (duplex H1‐H2) respectively, *n* = 3. Error bar, mean ± SD. B) The conversion efficiency N of DCHA‐E (blue line), DCHA‐F (red line), and CHA‐F (black line), respectively (E: electrochemistry method, F: fluorescence method), *n* = 3. Error bar, mean ± SD.

Based on results above and Figure S5 (Supporting Information), the concentrations of output resultants in DCHA (duplex H2‐H3) and CHA (duplex H1‐H2) corresponding to different concentrations of target miRNA‐21 were computed, respectively. As depicted in **Figure** **5**A, we could see that the concentration of duplex H2‐H3 in DCHA was all overtop that of duplex H1‐H2 in CHA with different concentrations of miRNA‐21, indicating that the DCHA could output much more resultants with the same input miRNA‐21 compared with CHA. Subsequently, the accurate conversion efficiency *N* (the concentration ratio between output resultant and input target miRNA‐21) of DCHA and CHA could be calculated (Table S2, Supporting Information). As shown in Figure 5B, the DCHA showed a hyper conversion efficiency in fluorescence method (DCHA‐F, 3.05×10^7^, red line), which is more than 2 orders of magnitude beyond the one of CHA (CHA‐F, 2.91×10^5^, black line), suggesting the dual catalysts indeed dramatically improved the performance of DCHA.

Moreover, for applying the DCHA in developing ultrasensitive electrochemical biosensor, the conversion efficiency of it in electrochemistry method (Figure S6, Supporting Information) was also computed and the results were shown in Figure 5B (blue line). From these results, we can see that the conversion efficiency of DCHA in electrochemistry method (DCHA‐E, 4.54×10^8^) was improved more than 1 orders of magnitude compared with the one in fluorescence method (3.05×10^7^), which could be ascribed to the high sensitivity of electrochemistry and the probably less interferences of the biphasic electrode operation.^[^ ^30^ ^]^ The above results illustrate that the DCHA has dramatically exceeded the CHA in both reaction rate and conversion efficiency and could be well applied in electrochemical biosensor construction for ultrasensitive detection of miRNA.

Otherwise, we compared the performances of DCHA with those of other typical isothermal amplification methods. As shown in **Table** **1**, the DCHA is endowed with the least reaction time, wide and high conversion efficiency even under room temperature, successfully addressing the challenges of these traditional nucleic acid amplifications: time‐consuming and low‐efficiency.

**Table 1:** Comparison of the developed DCHA with other amplification methods

Method	Temperature [℃]	Reaction time [h]	Efficiency	Ref.
NASBA	≈41	1.5–2	10^6^–10^9^	[31, 32]
E‐SDA	37	2	10^7^	[1, 33]
HRCA	60	1.5	10^9^	[34, 35]
PG‐RCA	60	1–3	≈60 Copies	[36, 37]
LAMP	60–65	< 1	10^9^	[38]
EXPAR	≈60	< 0.5	10^6^–10^8^	[39, 40]
HDA	37–65	0.5–2	10^7^	[41, 42]
MDA	30–37	8	10^6^	[43, 44]
RPA	37–42	0.5–1.5	10 Copies	[45]
pWGA	37	0.5–2	10^3^–10^8^	[46]
CHA	25	1.3	7.90–2.91×10^5^	[47, 48]
DCHA	25	0.3	1.89×10^3^–4.54×10^8^	This work

### 2.4 Application Evaluation of DCHA in Cancer Biomarker miRNA Ultrasensitive Assay

**Figure 6:**
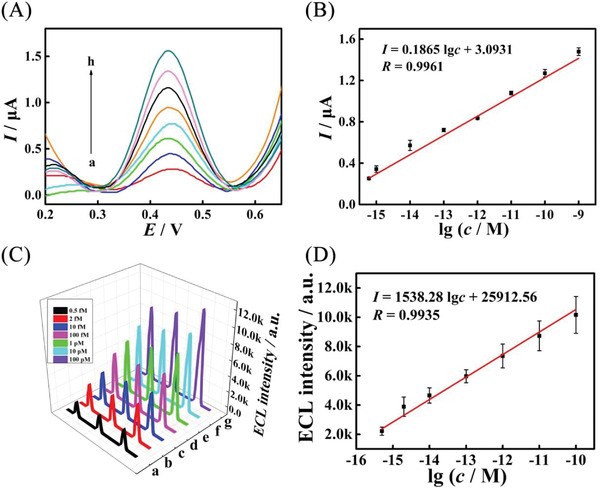
Application of DCHA in cancer biomarker miRNA ultrasensitive assay. A) SWV current responses of the biosensors to different concentrations of the target miRNA‐21: a) 0.6 × 10^−15^ m, b) 2.0 × 10^−15^ m, c) 10 × 10^−15^ m, d) 100 × 10^−15^ m, e) 1.0 × 10^−12^ m, f) 10 × 10^−12^ m, g) 100 × 10^−12^ m, h) 1 × 10^−9^ m; B) corresponding calibration plot for the SWV peak current versus l g *c*, *n* = 3. Error bar, mean ± SD. C) ECL intensity‐potential curves of the proposed biosensor incubated with miRNA‐21 concentrations of 0.5 × 10^−15^ m, 2 × 10^−15^ m, 10 × 10^−15^ m, 100 × 10^−15^ m, 1 × 10^−12^ m, 10 × 10^−12^ m, 100 × 10^−12^ m (from a to g). D) the calibration plot of ECL intensity with the logarithm of miRNA‐21 concentration, *n* = 3. Error bar, mean ± SD.

As a proof of concept, the DCHA was applied as high‐efficiency DNA signal magnifier to construct electrochemical and electrochemiluminescence (ECL) biosensor for the rapid and ultrasensitive detection of miRNA (the feasibility of biosensor construction was characterized in Figures S8–S10 and S12, Supporting Information). Under the optimal experimental conditions (Figures S2 and S11, Supporting Information), the proposed electrochemical biosensor was incubated target miRNA‐21 with different concentrations and evaluated by square wave voltammetry (SWV). As shown in **Figure** **6**A, the SWV current response gradually increased as the concentration of target increased from 0.6 × 10^−15^ m to 1 × 10^−9^ m and showed a well linear relationship with the logarithm of the miRNA‐21 concentration. As displayed in Figure 6B, the regression equation is expressed as *I* = 0.1865 lg *c* + 3.0931 with a *R* of 0.9961. According to the 3*σ* rule, a LOD of 0.34 × 10^−15^ m for miRNA‐21 was calculated out (Table S3, Supporting Information). Moreover, as shown in Figure 6C, the signals of the developed ECL biosensor exhibited monotonically increasing with elevated concentrations of target miRNA‐21 from 0.5 × 10^−15^ m to 100 × 10^−12^ m. As illustrated in Figure 6D, the developed biosensor manifested an outstanding linear relationship between the ECL intensity and the logarithm of various concentrations miRNA‐21. The corresponding regression equation was *I* = 1538.28 lg *c* + 25912.56 with a R of 0.9935 and the pretty lower LOD down to 0.19 × 10^−15^ m (Table S3, Supporting Information). Significantly, as illustrated in **Table** **2**, compared with other biosensors, the developed biosensor had a wider linear range and an extremely lower detection limit, which could be due to the elaborated DCHA with hyper conversion efficiency.

**Table 2:** Comparison of the biosensor developed by DCHA with other miRNA detection methods

Analytical methods	Linear range	Detection limit	Ref.
Fluorescence	1 × 10^−12^ m to 10 × 10^−9^ m	0.35 × 10^−12^ m	[49]
Photoacoustic Imaging	10 × 10^−12^ m to 100 × 10^−9^ m	11.69 × 10^−12^ m	[50]
Electrochemiluminescence	10 × 10^−15^ m to 0.1 × 10^−9^ m	6.6 × 10^−15^ m	[51]
Chronocoulometry	2.0 × 10^−15^ m to 1.0 × 10^−9^ m	2.0 × 10^−15^ m	[52]
Electrochemical	0.14 × 10^−9^ m to 10 × 10^−9^ m	40 × 10^−12^ m	[53]
Electrochemical	1 × 10^−12^ m to 1 × 10^−9^ m	10 × 10^−12^ m	[54]
Electrochemical	0.6 × 10^−15^ m to 1 × 10^−9^ m	0.34 × 10^−15^ m	This work
Electrochemiluminescence	0.5 × 10^−15^ m to 100 × 10^−12^ m	0.19 × 10^−15^ m	This work

In addition, the reproducibility, selectivity and stability of the proposed biosensor based on the DCHA were also validated. As the results of reproducibility study illustrated in Figure S14A (Supporting Information), the responses of four prepared biosensors to miRNA‐21 (10 × 10^−12^ m) shows a low relative standard deviation (RSD) of 6.31%, moreover, a set of electrodes with same miRNA‐21 (10 × 10^−12^ m) were also detected after 15 days, and the RSD is only 5.05%. Subsequently, the prepared biosensor displayed an obvious response only in the presence of target miRNA‐21 even with low concentration (selectivity, Figure S14B, Supporting Information). As for the stability (Figure S14C, Supporting Information), the SWV current intensity of the biosensor to miRNA‐21 (10 × 10^−12^ m) only changed from 94.5% to 105.0% with the RSD = 3.86%. The results above successfully demonstrated the biosensor based on DCHA possesses outstanding reproducibility, selectivity, and stability.

Impressively, as shown in Figure S15 (Supporting Information), the capacity of the elaborated biosensor for miRNA‐21 detection was further investigated with total RNA extraction solutions from human cancer cell lines MCF‐7 and HeLa, and the results (Figure S15, Supporting Information) exhibited a significant expression level of target miRNA‐21 from MCF‐7, whereas in HeLa cells, a relatively low expression level compared to MCF‐7 were noticed, which were in accordance to previous reports.^[^ ^55^, ^56^ ^]^ Next, the DCHA was further validated in living MCF‐7 cells and compared with traditional CHA strategies using the total internal reflection fluorescence microscope (TIRFM). As the results displayed in Figure S16 (Supporting Information), the fluorescence response of DCHA system in MCF‐7 reached a stronger saturation in a much shorter time (Figure S16A, maximum at 30 min, Supporting Information) compared with that of traditional CHA strategy (Figure S16B, maximum at 90 min, Supporting Information), further demonstrating that the DCHA system can still maintain high‐speed and hyper‐efficiency performance when monitoring miRNA in living cells.

## 3 Conclusion

In summary, unlike the traditional nucleic acid signal amplification methods, the dual‐catalyst hairpin assembly (DCHA) we proposed realizes the recycle of two catalysts (target miRNA and H1) by introducing the well‐designed H3, which keeps the intermediate product duplex H1‐miRNA with an almost high concentration therefore achieving high reaction speed and hyper conversion efficiency. Benefiting from these advantages, the DCHA is successfully applied as high‐efficiency DNA signal magnifier in the rapid and ultrasensitive detection of miRNA‐21, which carves out a span‐new way to design potential strategy for the biosensing assay and clinic diagnose. Otherwise, the DCHA also proposes a creative insight to explore the inherent property of nucleic acid amplification for advancing its superiority and applicability in diagnostic applications, biological research, nanobiotechnology, and so on.

## 4 Experimental Section

### Chemical and Materials

Gold chloride (HAuCl_4_•4H_2_O), 6‐Mercaptohexan‐1‐ol (MCH), and Tris (2‐carboxyethy) phosphine hydrochloride (TCEP) were purchased from Sigma (St. Louis, MO). The HPLC‐purified miRNAs and DNAs were supplied by Sangon Inc. (Shanghai, China) and Tsingke (Beijing, China), respectively, and Table S1 (Supporting Information) showed the sequences of the oligonucleotides used in the experiment. Tris‐HCl buffer (20 × 10^−3^ m Tris, 140 × 10^−3^ m NaCl, 1.0 × 10^−3^ m MgCl_2_, 5 × 10^−3^ m KCl, 1.0 × 10^−3^ m CaCl_2_, 1.0 × 10^−3^ m TCEP, pH = 7.4) was harnessed to dilute oligonucleotides. 5xTBE buffer (250 × 10^−3^ m Tris, 250 × 10^−3^ m H_3_BO_3_, 10 × 10^−3^ m EDTA, pH 8.0) and acrylamide (29:1, 40%), ammonium persulfate (APS), tetraethylenediamine (TEMED), and Gel‐red dye obtained from Dingguo Biotechnology Co., LTD (Chongqing, China) was used to perform PAGE experiments. Dulbecco's Modified Eagle Medium (DMEM), sterile PBS, and Lipofectamine Reagent‐3000 were provided by Thermo Fisher Scientific Inc. (Shanghai, China) and Hoechst 33342 (nuclear staining) was purchased from Beijing solarbio science & technology co., ltd. for cell experiments. Phosphate buffered solution (PBS) buffer (100 × 10^−3^ m Na_2_HPO_4_, 100 × 10^−3^ m KH_2_PO_4_, 100 × 10^−3^ m KCl, pH 7.0), and [Fe(CN)_6_]^3−/4–^ solution (20 × 10^−3^ m Tris‐HCl, 5 × 10^−3^ m K_3_[Fe(CN)_6_], 5 × 10^−3^ m K_4_[Fe(CN)_6_], pH 7.4) were prepared to carry out the performance measurements of the electrochemical biosensor. The DNA strand H2 was labeled with RuBpy (Ru) when as an ECL signal tag Electrochemiluminescence (ECL) biosensor construction according to previous literature.^[^ ^57^ ^]^

### Apparatus and Measurements

Cyclic voltammetry (CV), electrochemical impedance spectroscopy (EIS), and SWV were accomplished by using a CHI760E electrochemical workstation (CH Instruments, Shanghai, China) with a three‐electrode arrangement. PAGE was conducted by a Bio‐Rad imaging system (Hercules, CA). The fluorescence experiments were carried out with an F‐2500 fluorescence spectrophotometer (Hitachi, Tokyo, Japan) (EX slit 5 nm, EM slit 10 nm, Voltage 700 V). Fluorescence imaging of living cells was used by an Olympus IX‐81 microscope equipped with a Total Internal Reflection Fluorescence (TIRF) microscopy (Olympus, Japan). The CV and EIS signal of the biosensor establishment were processed in 2 mL 5 × 10^−3^ m [Fe(CN)_6_]^3−/4−^ solution (scanning potential: −0.2–0.6 V, scan rate: 100 mV s^−1^). And the SWV was conducted in 0.1 m PBS with the potential from 0.2 to 0.7 V (Amplitude 25 mV, Frequency 15 Hz, and Quiet time 2 s) and the proposed ECL biosensor was detected in 2 mL PBS buffer (pH = 7.4) including 15 × 10^−3^ m TPrA under the scanning potential from 0 to 1.25 V with a scan rate of 200 mV s^−1^, which was applied to evaluate the performance of the proposed electrochemical and ECL biosensor under the optimal experimental conditions.

### Preparation of the Hairpin H1, H2, and H3

The DNA strands H1, H2, and H3 in Tris‐Hcl buffer (7.4) were, respectively, heated to 95 °C for 10 min and then cooled down to room temperature over 30 min to form the hairpin H1, H2, and H3. The prepared hairpin DNAs were used in subsequent experiments.

### Assembly of the Elaborated Biosensor

First, the bare glassy carbon electrode (GCE, 4 mm in diameter) was polished carefully with alumina slurry (0.3 and 0.05 µm), following by ultrasonic treatment with ethanol and distilled water. After that, the electrode was immersed into a HAuCl_4_ aqueous solution (1%) and electrodeposited at −0.2 V for 30 s to coat a layer of gold particles (depAu), and immersed into HPtCl_4_·6H_2_O (1%) solution and electrodeposited at −0.25 V for 30 s to attain platinum nanoparticles modified layer (depPt) for electrochemical and ECL biosensor construction separately.

Subsequently, 10 µL of the hairpin H3 solution (2.0 × 10^−6^ m) was dropped onto the surface of the electrode (depAu/GCE) and incubated overnight at room temperature. After the electrode was rinsed with Tris‐Hcl buffer to remove nonspecifically adsorbed DNA, the electrode was incubated with MCH (5 × 10^−3^ m) for 30 min to block the remaining active sites of electrode and then rinsed by ethanol and distilled water for three times.

### DCHA on the Electrode Surface

First, the target with different concentrations was mixed with the prepared hairpin H1 and H2 in Tris‐Hcl buffer. Then 10 µL of the mixture above was dropped on the electrode (MCH/H3/depAu/GCE) and incubated under the optimal conditions.

### Cell Culture and Total RNA Extraction

The HeLa and MCF‐7 cancer cells applied in this study were obtained from the cell bank of the Chinese Academy of Sciences (Shanghai, China). According to the manufacturer's instructions, the cancer cells were cultured in RPMI 1640 medium (Thermo Scientific Hyclone, USA) with the addition of 10% fetal calf serum (FCS), 100 U mL^−1^ penicillin and 100 µg mL^−1^ streptomycin at 37 °C in 5% CO_2_ incubator. Then, the total RNA samples were extracted from the cancer cells by using the Trizol Reagent Kit (Sangon, Inc., Shanghai, China) on the basis of operating instructions. At last, the obtained cellular extracts were stored at −80 °C when not in use.

### Cell Transfection for the DCHA System

The procedures for miRNA imaging using TIRFM were as follows. At first, the MCF‐7 cells were introduced into a 35 mm^2^ Petri dish. Immediately, they were respectively cultured in DMEM medium with a humidified atmosphere (5% CO_2_) at 37 °C for 24 h to reach 80% cell anchorage‐dependent rate, and then washed with sterile PBS. After that, the 100 µL solutions containing Lipofectamine Reagent‐3000 (5 µL) and hairpin DNA substrates (1 × 10^−6^ m) was mixed sufficiently at room temperature for 5 min and incubated into MCF‐7 cells Petri dish with a humidified atmosphere (5% CO_2_) at 37 °C for 2 h. Subsequently, the cells were stained with a Hoechst 33342 solution for 15 min and washed three times with sterile PBS. After that, the cells were incubated with 1 mL of fresh DMEM medium at room temperature before fluorescence imaging.

### PAGE

First, distilled water (10 mL), 5xTBE buffer (5 mL), acrylamide (40%, 10 mL), APS (10%, 200 µL), and TEMED (15 µL) were mixed successively while being stirred constantly. 1 min later, the mixed solution was added into the glass mold and kept for about 5 min under room temperature for ultimate 16% polyacrylamide gel formation. Subsequently, after the DNA samples were mixed with the DNA‐loading buffer (volume ratio 5:1), the dynamic DNA‐assembled products were characterized by PAGE on the freshly prepared 16% polyacrylamide gel in 1× TBE buffer at 60 mA.

### Operation of the Biosensing Platform for the MiRNA‐21 Assay in Cancer Cell Lysates

Primarily, the cancer cell lysates from different number of cells were mixed with the prepared hairpin DNAs H1 and H2 in Tris‐HCl buffer (pH = 7.4). Subsequently, the 10 µL of the obtained mixture above was dropped onto the electrode surface modified with hairpin DNA H3 (MCH/H3/depAu/GCE) and incubated under optimal conditions. After the reaction was finished, the electrode surface was washed by Tris‐HCl buffer (pH = 7.4) for three times at least for subsequent electrochemical measurements.

## Conflict of Interest

The authors declare no conflict of interest.

## Supporting information

Supporting Information

## Data Availability

Research data are not shared.
