# Supplementary material for: Programmable High‐Speed and Hyper‐Efficiency DNA Signal Magnifier
Source: Adv Sci (Weinh). 2021 Dec 16;9(4):2104084. doi: 10.1002/advs.202104084 (PMC8811820; doi:10.1002/advs.202104084)
Supplement: Supplementary file 1 — Supporting Information [file ADVS-9-2104084-s001.pdf]

ADVANCED SCIENCE
Open Access

Supporting Information

for *Adv. Sci.*, DOI: 10.1002/advs.202104048

Programmable High-speed and Hyper-efficiency DNA Signal Magnifier

*Xiao-Long Zhang, Yang Yin, Shu-Min Du, Ling-Qi Kong, Zhe-Han Yang, Yuan-Yuan Chang, Ya-Qin Chai, and Ruo Yuan**

Supporting Information

Programmable High-speed and Hyper-efficiency DNA Signal Magnifier

Xiao-Long Zhang, Yang Yin, Shu-Min Du, Ling-Qi Kong, Zhe-Han Yang, Yuan-Yuan Chang, Ya-Qin Chai, and Ruo Yuan*

Key Laboratory of Luminescence Analysis and Molecular Sensing (Southwest University), Ministry of Education, College of Chemistry and Chemical Engineering, Southwest University, Chongqing 400715, PR China

*Corresponding author. Email: yuanruo@swu.edu.cn

Supplementary Text

Figs. S1 to S16

Tables S1 to S3

References (1 to 13)

1. Supplementary Text

Characterization of the Nucleic Acid Hybridizations in DCHA and CHA

The reaction mechanism of DCHA and CHA was characterized by PAGE. As shown in Figure S1, the obvious bands in lanes 1−4 correspond to miRNA-21, hairpin H1, hairpin H2, and hairpin H3 separately. After the miRNA-21 was mixed with hairpin H1, in comparison with lane 1 and lane 2, a new band with slower migration (duplex H1-miRNA-21) could be observed and the band corresponding to miRNA-21 vanished (lane 5), suggesting the hairpin H1 was opened and hybridized with the target. Then, after the H2 was added into the mixture of miRNA-21 and H1, another obvious band with slower migration (lane 6) compared with the one of duplex H1-miRNA-21, which corresponded to the duplex H1-H2 could be noticed, indicating the H2 successfully hybridized with duplex H1-miRNA-21 and displaced the miRNA-21 comparing with lane 5. Moreover, with the introduction of hairpin H3 into the mixture above (lane 6), the band corresponding to the duplex H1-H2 was dilute and an new obvious band with very low migration could be noticed (lane 7), verifying the successful displacement reaction between the hairpin H3 and duplex H1-H2 and the hybridization of H2 and H3 (duplex H2-H3). These observations strongly indicated these nucleic acid hybridizations in DCHA and CHA could be successfully proceeded.

Optimization of the Reaction Temperature of DCHA.

Since the reaction temperature is of great importance for the nucleic acid amplification, we optimize the reaction temperature of DCHA. As shown in Figure S2, the fluorescence response gradually decreased with the elevated reaction time of the DCHA, indicating the reaction rate and conversion efficiency of DCHA under different temperature were almost the same from 25

^{o}C to 40 ^{o}C. However, under 45 ^{o}C, the conversion efficiency of DCHA decreased obviously which was due to the background fluorescence intensity from the unwinding of the DNA duplex. As a result, considering the stability and reactivity of DNA, the reaction temperature of DCHA was fixed at 25 ^{o}C (room temperature).

Quantification of intermediate product DNA-21-H1 in DCHA and CHA

To better quantify the intermediate product DNA-21-H1 in the reaction process of DCHA and CHA, we computed the specific concentration of it (the total concentration of dissociative DNA-21 and DNA-21-H1 are 1 μM) based on Figure 1A and the relationship between fluorescence intensity and concentration of the fluorophore FAM (Figure S5). As the results shown in Figure S3, the concentration of F-DNA-21-B-H1 in CHA continually decreased from 0.91 μM to 0.18 μM (0-1500 s), however, the one in DCHA almost maintained a relatively high value from 0.42 μM to 0.82 μM, the ultimate concentration of which was more than four times the one in CHA (2500 s). These results illustrated the method we proposed could indeed maintain the intermediate product in high concentration while the one in traditional methods decreased constantly.

Background Leakage Comparison Between DCHA and CHA

Considering the inherent problem of signal leakage in traditional CHA, we studied the change of blank fluorescence signal in DCHA (H1 1 μM, F-H2 1 μM, B-H3 1 μM) and CHA (B-H1 1 μM, F-H2 1 μM) for 1 h. As displayed in Figure S4, with the time increased, the fluorescence intensity of DCHA just decreased to a slightly lower value when compared with that of CHA,

suggesting acceptable background leakage of DCHA which almost plays negligible effect on the performance enhancing.

Relationship between the Signal Intensity and the Signal Tag Concentration in Fluorescence and Electrochemisty

To measure the practical conversion efficiency N of DCHA and CHA in fluorescence, we firstly carried out the relationship of the fluorescence response and the concentration of the fluorescent tag FAM labeled on the DNA. As shown in Figure S5A, with the concentration of FAM increased, the fluorescence intensity increased obviously and displayed a fine liner relationship with the concentration. And the corresponding regression equation was $Fi = 46.43 + 2849.79\ c$ (from 0.1 μM to 1 μM, R=0.9818).

Subsequently, in view of the performance of DCHA when used in electrochemical biosensor construction (Scheme 1), we further measure the conversion efficiency of DCHA in electrochemistry. The relationship of the current response of biosensor and the concentration of the quantified independent H2-H3 (H2 was labeled with electroactive tag ferrocene) was carried out based on the SWV. As shown in Scheme 1, the biosensing platform was directly incubated with different concentrations of H2-H3, which can be captured by the electrode modified depAu through forming the Au-S bond and cause an obvious current response on the electrode surface. As shown in Figure S6A, the SWV current response increased as the concentration of H2-H3 from 50 nM to 2 μM and displayed a fine linear relationship with the concentration (Figure S6B). The corresponding regression equation was $I = 0.0593 + 0.7148\ c$ (R=0.9991, I and c represented the SWV current response and H2-H3 concentration, separately; unit of c was M).

Measurement of the rate constant *k* of the DCHA and CHA

As reported in the previous literatures,[1] at sufficiently low concentration, the kinetics of toehold strand displacement reaction (TSDR) are well approximated by bimolecular reactions with second-order rate constants. Assuming the reactants quickly resolve into products,[2] the reaction process of DCHA and CHA which are developed based on TSDR could be respectively modeled as

$$\mathrm{H2} + \mathrm{H3} \xrightarrow[k_1]{\text{DNA-21,H1}} \mathrm{H2H3} \qquad (1)$$

$$\mathrm{H1} + \mathrm{H2} \xrightarrow[k_2]{\text{DNA-21}} \mathrm{H1H2} \qquad (2)$$

where k_1 and k_2 represent the second-order rate constant of DCHA and CHA separately. According to the related kinetic study,[3,4] the k_1 and k_2 could be computed by the equation

$$t_{1/2} = \frac{1}{kc_0} \qquad (3)$$

where the $t_{1/2}$ represents half-period and the c_0 is the initial concentration of reactants. Specially, we mixed DNA-21, H1, FN-H2 labeled FAM and BHQ1, and H3 for DCHA and mixed the DNA-21, H1, FN-H2 labeled FAM and BHQ1 for CHA to monitor the fluorescence intensity of them during the reaction process (the fluorescence of FAM labeled on 5' of H2 could regain while the FN-H2 was opened, Excitation (EX) 492 nm, Emission (EM) 528 nm, EX slit 5 nm, EM slit 5 nm, Voltage 700 V). After converting the fluorescence intensity into *P* (the cumulative probability (*P*) represents the degree of conversion, which was calculated by dividing the number of satellite leaving events up to a given time, by the total number of events observed during the experimental time course), as the results depicted in Figure S7, the $t_{1/2}$ of DCHA and CHA were

260 s and 685 s separately. Then based on the equation (3), the rate constant of k_1 and k_2 can be estimated as 3.85×10^3 $M^{-1}s^{-1}$ and 1.46×10^3 $M^{-1}s^{-1}$ respectively.

Electrochemical Characterization of the Designed Biosensor

The CV and EIS were used to characterize the stepwise fabrication of the biosensor. As displayed in Figure S8A (CV), a pair of well-defined redox peaks (curve a) of the bare GCE could be noticed. When the GCE was electrodeposited with the AuNPs, the redox currents increased (curve b), for the excellent conductivity of the AuNPs. Subsequently, with the immobilization of hairpin H3, the redox currents decreased obviously (curve c), because of the repulsion effect between $[Fe(CN)_6]^{3-/4-}$ and the DNA phosphate backbone with negatively charge. And when the MCH was self-assembly on the electrode, the redox currents decreased slightly (curve d), further meaning that the electrode surface almost has been occupied with the H3. Moreover, after the DCHA was triggered by the target on the modified electrode surface, the redox currents decreased (curve e) owing to the hybridization of H2 and H3 which carried more negative charge onto the electrode surface.

Then as shown in Figure S8B (EIS), a small semicircle diameter and a long tail denoting diffusion could be observed, for the well conductivity of the bare GCE (curve a). And we can find a nearly straight line after the depAu was electrodeposited onto the bare GCE (curve b), because of the large surface area and the excellent conductivity of the depAu. Next, with the self-assembly of more DNA strands and MCH onto the electrode, a dramatically increasing trend of Ret (curves c, d, e) could be noticed, which was ascribed to the increased steric hindrance from the stack of DNA strands and MCH. These EIS results were in accordance with the CV results which were observed above, further demonstrating the successful fabrication of this biosensor.

Moreover, the electroactive area of the modified electrode (depAu/GCE) and the density of H3 on the modified electrode were also determined by cyclic voltammetry (CV) and chronocoulometry (CC). Firstly, CV studies were carried out according to the previous works.[5,6] The cyclic voltammograms (CVs) were operated[7] at different potential scan rates (10, 30, 50, 70, 90, 110, 130, 150, 170, 190, and 210 mV/s) in 5 mM $[Fe(CN)_6]^{3-/4-}$ solution, as shown in following Figure S9A. The linear regression for the peak current (Ip) *versus* the square root of scan rate ($v^{1/2}$) is presented in following Figure S9B. According to the slope S and the Randles-Sevcik equation:

$$I_p = (2.69\times10^5)n^{3/2}AD^{1/2}cv^{1/2} \qquad (4)$$

the electroactive area A can be expressed as eqn (2).

$$A = S/(2.69\times10^5)n^{3/2}D^{1/2}c \qquad (5)$$

where n is the number of electrons involved (n=1), D is the diffusion coefficient (6.72×10^{-6} cm^2 s^{-1}, 25 ℃), and c is the concentration of ferricyanide (5 mM). Thus the electroactive area A of the modified electrode was calculated as 19.3 mm^2.

Secondly, according to the previous report,[8] the CC studies were performed and the density of H3 Γss was calculated by using the following eqn (6) and (7).

$$\Gamma_{ss} = (Q_{ss}N_A/nFA)(z/m) \qquad (6)$$

$$Q_{ss} = Q_{total} - Q_{dl} \qquad (7)$$

And N_A is Avogadro's number, n is the number of electrons, A is the electroactive area, m is the number of nucleotides in the DNA and z is the charge of the redox molecule. Q_{total} and Q_{dl} are obtained from the plot of the charge (Q) versus the square root of scan time ($t^{1/2}$), as shown in following Figure S10. So the density of H3 on depAu/GCE in this work is 8.02×10^{11} molecules cm^{-2}.

Optimization of Experimental Conditions

Because the accuracy and the sensitivity of this proposed biosensor largely depend on the efficiency of the DCHA, three experimental parameters containing the immobilization concentration of capture probe H3, the concentration ration of H2 and H3, and the reaction time of DCHA. Firstly, as shown in Figure S11A, the current response gradually increased with the elevated immobilization concentration of the capture probe H3 from 0.5 μM to 3.0 μM, since more signal tag DNAs were captured by H3 on the sensor surface. However, a slight decrease of the current response should be noted when the immobilization concentration were beyond 2.0 μM possibly owing to the improved steric hindrance of high immobilization concentration of H3. As a result, the immobilization concentration of the H3 at 2.0 μM was set for the subsequent experiments. Then, as shown in Figure S11B, with the concentration ratio of H2 and H3 increased from 1 to 3, the current response increased and then slightly decreased, and reached the maximum at 2.0, showing the optimal concentration ratio for DCHA was 2.0. Next, we examined the effect of the reaction time of the DCHA, as displayed in Figure S11C, the current response increased with a dilated reaction time from 5 to 30 min and thereafter reached a plateau, suggesting the reaction equilibrium after 20 min. Thus, the reaction time of DCHA was selected at 20 min. From these results, the immobilization concentration of capture probe H3, concentration ratio of H2 and H3, and the reaction time of DCHA were therefore fixed at 2.0 μM, 2.0, and 20 min respectively.

Comparison of the Current Responses of the proposed Biosensor under Different conditions

In order to prove that only the presence of target miRNA and H1 can achieve the DCHA, SWV was used to study the electrochemical signal of the developed biosensor under different conditions. As shown in Figure S12, no SWV current response (curve a) was observed without the presence of target miRNA, H1 and Fc-labeled H2 owing to the absence of Fc in the buffer. And the incubation of biosensor with H2 and either target miRNA (curve b) or H1 (curve c) alone caused negligible current changes owing that the Fc-labeled H2 couldn't be captured on the electrode surface without the triggered DCHA. When both the target miRNA and H1 were mixed with Fc-labeled H2 and incubated with the biosensor, an distinct current response (curve d) was observed, suggesting that the DCHA triggered by both the target miRNA and H1 could indeed export amounts of Fc-labeled H2 to be captured by the biosensing platform, thus enhancing the electrochemical signal.

Limit of Detection Calculation for the miRNA Biosensing

On the basis of IUPAC definition, we have calculated the limit of detection according to equation (1) and the calibration plot (2) where x_B is the SWV intensity of blank sample and s_B is the standard deviation of blank samples, and IUPAC suggests that $k = 3$ allows a confidence level of 99.86%.[9]

$$I_L = x_B + k \times s_B \qquad (8)$$

$$I = 0.1865 \lg c + 3.0931 \qquad (9)$$

In this paper, specifically, the SWV measurements for blank samples were executed with ten parallel tests, which exhibited an average electrochemical signal intensity of blank sample of 0.14 μA and a standard deviation (SB) of blank sample with 2.34×10^{-8} μA. On account of this, the electrochemical signal intensity of detection limit (I_L) is estimated to be 0.21 μA. Under the

equation of the calibration (9), the estimated detection limit is calculated to be 0.34 fM. Similarly, the detection limit of fluorescence biosensor based on CHA and DCHA, and ECL biosensor based on DCHA for miRNA-21 detection are calculated to be 0.36 pM, 6.21 fM, and 0.19 fM, respectively (Table S3).

Reproducibility, Selectivity, and Stability of the Biosensor

In order to further study the performance of this proposed biosensor with mismatches comprehensively, the reproducibility, selectivity and stability of the biosensing platform were validated. To confirm the reproducibility of the biosensing platform, the necessary index for a real biomarker detection, four prepared biosensors with 10 pM miRNA-21 were studied under the same determination condition. As displayed in Figure S14A, a relative standard deviation (RSD) of 6.31% was achieved. Moreover, a set of electrodes with same miRNA-21 (10 pM) were detected after 15 days, and the RSD is 5.05%. The remarkable results indicate that the combination of the evolved DCHA for biomarker detection presented a desirable reproducibility.

To assess the selectivity of this biosensor, five interference agents including miRNA-126, miRNA-141, miRNA-155, and miRNA-182-5P were evaluated under the same experimental condition. As shown in Figure S14B, the obvious current response of the electrode could be observed only with miRNA-21 (10 pM, f). Oppositely, in the presence of the interference agents including miRNA-126 (1 nM, b), miRNA-141 (1 nM, c), miRNA-155 (1 nM, d), and miRNA-182-5p (1 nM, e), the current responses were negligible. Besides, the mixed analytical solution containing 10 pM of target miRNA-21 was also analyzed with the biosensing system, exhibiting an obvious current response as expected (g). These results above demonstrated the high selectivity of the biosensor.

The stability, which was critical to its practical application especially in a complicated biomedical environment, was evaluated by successive scans of the sensing platform with detection of 10 pM miRNA-21 for 15 times. As displayed in Figure S14C, when compared with the initial electrochemical response, the SWV current intensity only changed from 94.5% to 105.0% with the RSD=3.86%, indicating an acceptable stability of our biosensing surface.

Real Application of the Biosensor in Cancer Cells

The capacity of the proposed biosensor for determination of miRNA-21 in real biological samples was investigated by taking the total RNA extraction solutions from the human cancer cell lines (MCF-7 and HeLa). As shown in Figure S15, the SWV response gradually increased as the cell numbers increased, exhibiting a significant expression level of target miRNA-21 from MCF-7, whereas in blank sample and HeLa cells, no obvious SWV responses were noticed, indicating a relatively low expression level compared to MCF-7. The above results were consistent to previous research,[10,11] further suggesting this quantification of miRNA-21 in cancer cells possesses the key potential for clinical diagnosis of cancers.

The Total Internal Reflection Fluorescence Microscope (TIRFM) Imaging of miRNAs in Living MCF-7 Cells

Having demonstrated the high performance of the DCHA to accurately quantify miRNA-21 analyte in vitro, we then applied the system to image and monitor miRNA-21 in living cells using the total internal reflection fluorescence microscope (TIRFM). MCF-7 cells, which are well known to overexpress miRNA-21,[12] were chosen to investigate the capability of the DCHA-mediated intracellular miRNA-21 imaging strategy compared with traditional CHA strategies. As

the results shown in Figure S16, with the reaction time increased, the FAM fluorescence (green) of DCHA system increased fast and reached saturation at only 30 min (Figure S16A), whereas in the traditional CHA system, the FAM fluorescence increased slowly and reached a weaker saturation after 90 min (Figure S16B). Additionally, the slightly decreased FAM fluorescence of both DCHA and CHA after 120 min was ascribed to the degradation of the hairpin oligonucleotide probes in the intracellular environment.[13] Considering these results, it can be concluded that the DCHA system could convert the miRNA in living cells into more resultants within much shorter times in comparison with traditional CHA strategy, demonstrating that the DCHA system can still maintain high-speed and hyper-efficiency performance when monitoring miRNA in living cells.

2.Supplemental Tables and Figures

2.1 Supplemental Figures

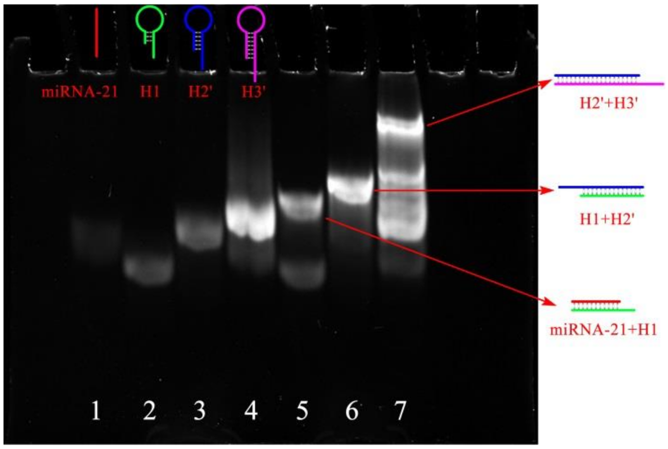

Figure S1. Nondenaturing PAGE characterization of DCHA and CHA. Lane 1, miRNA-21 (4 μM); lane 2, H1 (2 μM); lane 3, H2' (2 μM); lane 4, H3' (2 μM); lane 5, microRNA-21 (2 μM) and H1 (2 μM); lane 6, microRNA-21 (2 μM), H1 (2 μM), and H2' (2 μM); lane 7, miRNA-21 (2 μM), H1 (2 μM), H2' (2 μM), and H3' (2 μM) (PAGE 16%, 60 mA, 120 min).

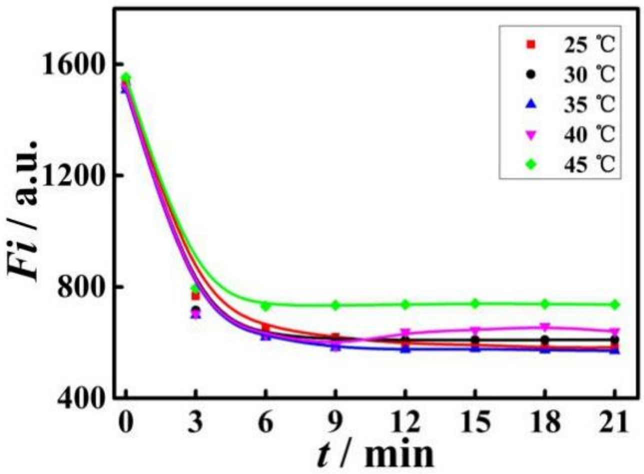

Figure S2. Optimization of reaction temperature of the DCHA. The fluorescence response of DCHA under different reaction temperature (25 °C, 30 °C, 25 °C, 40 °C, 45 °C), n=3. Error bar, mean ± SD.

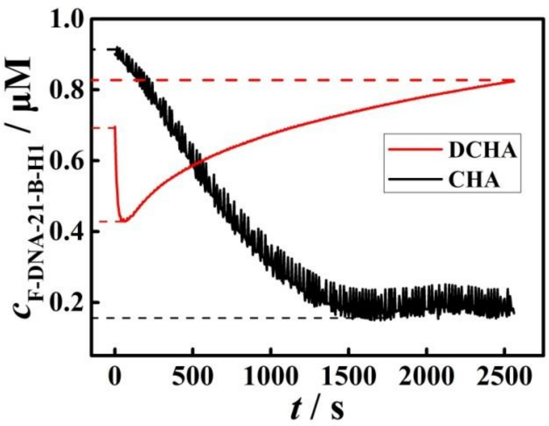

Figure S3. Dynamical monitoring of the intermediate product F-DNA-21-B-H1. Concentration Change of the intermediate product F-DNA-21-B-H1 in the reaction process of DCHA and CHA (the initial concentration of DNA-21 and H1 were 1 μM).

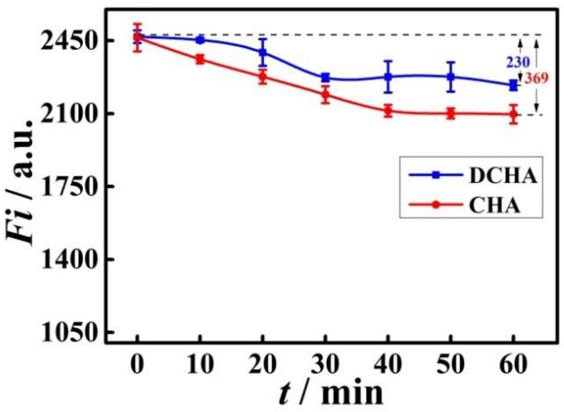

Figure S4. Background leakage monitoring. Comparison of the background leakage between DCHA and CHA in fluorescence signal response (EX slit 5 nm, EM slit 10 nm, Voltage 700 V), n=3. Error bar, mean ± SD.

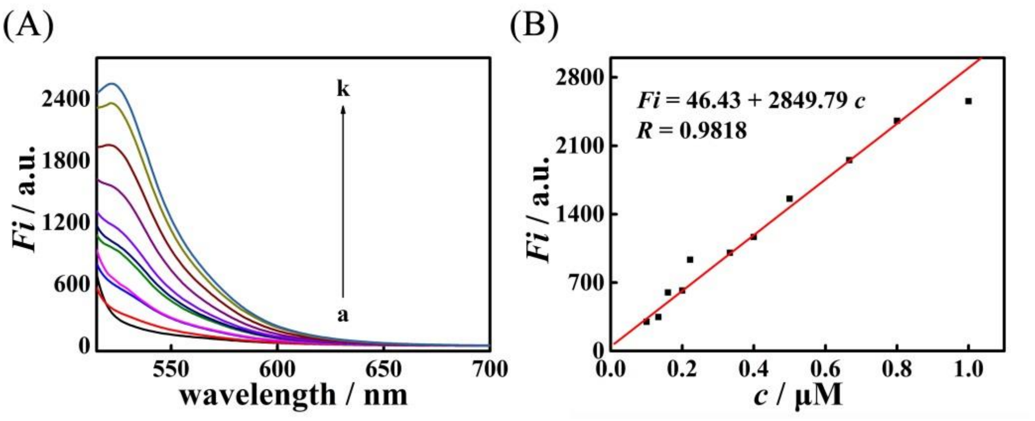

Figure S5. Fluorescence response to the fluorophore FAM. (A) Fluorescence intensity of different concentrations of the FAM: (a) 0.1 μM, (b) 0.133 μM, (c) 0.16 μM, (d) 0.2 μM, (e) 0.222 μM, (f) 0.333 μM, (g) 0.4 μM, (h) 0.5 μM, (i) 0.667 μM, (j) 0.8 μM, (k) 1.0 μM and (B) corresponding calibration plot for the fluoresence intensity *vs* *c*, n=3. Error bar, mean ± SD.

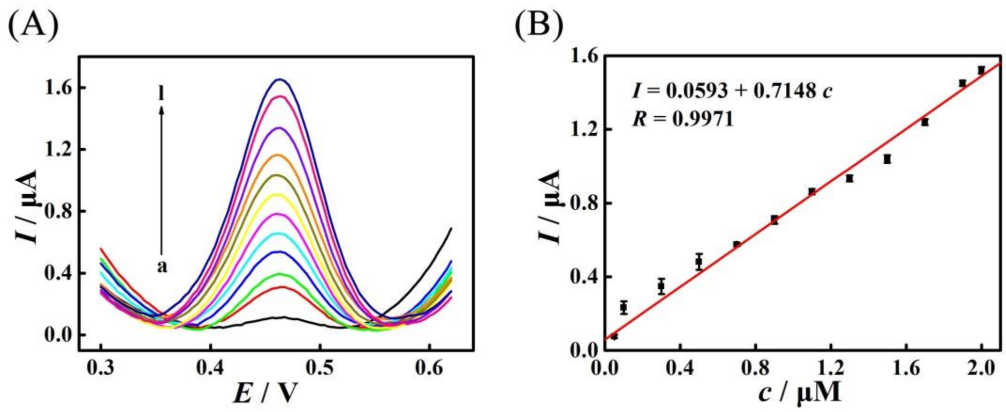

Figure S6. Current response of the biosensor to the electroactive material Fc. (A) SWV current responses of the biosensing platform to different concentrations of the H3-H2: (a) 50 nM, (b) 0.1 μM, (c) 0.3 μM, (d) 0.5 μM, (e) 0.7 μM, (f) 0.9 μM, (g) 1.1 μM, (h) 1.3 μM, (i) 1.5 μM, (j) 1.7 μM, (k) 1.9 μM, (l) 2.0 μM, and (B) the calibration plot for the SWV peak current *vs c* (*c* represents the miRNA-21 concentration), n=3. Error bar, mean ± SD.

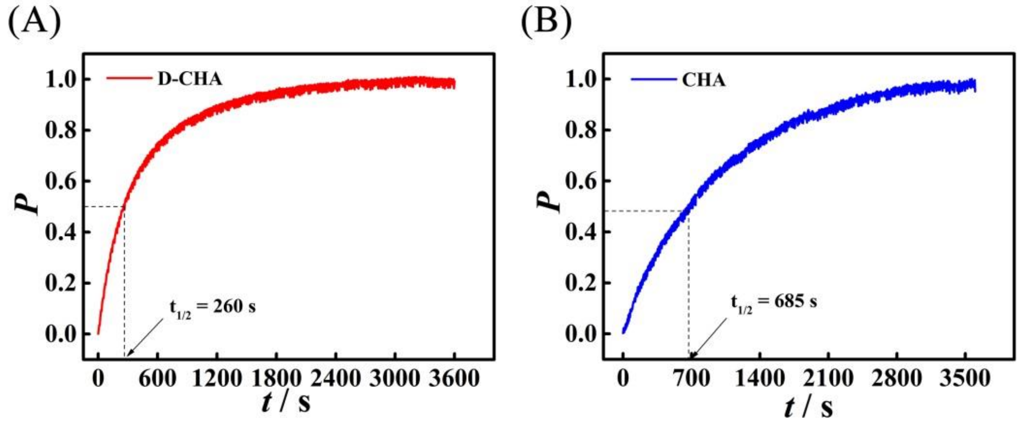

Figure S7. Measurement of the rate constant k of the D-CHA and CHA. Cumulative probability converted by fluorescence signal over time measured for (A) DCHA and (B) CHA. The concentrations of all DNA strands was 1 μM.

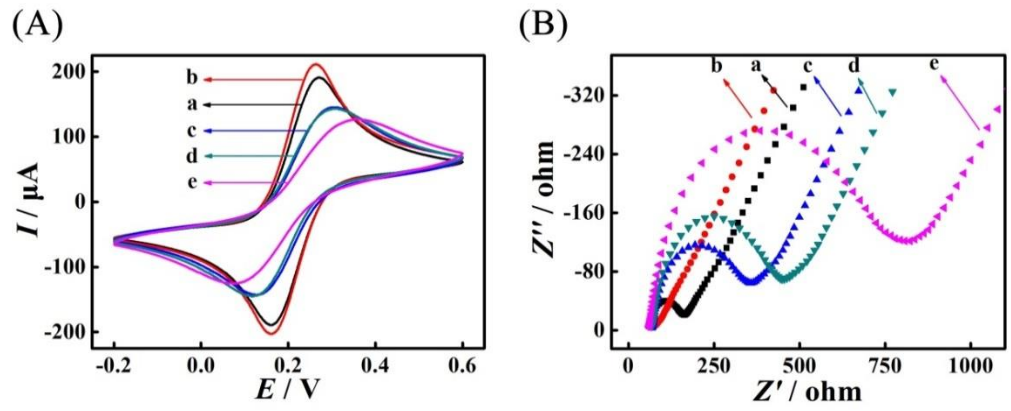

Figure S8. Electrochemical characterization of the prepared biosensor. (A) Typical CV responses and (B) EIS responses of different modified electrodes: (a) bare GCE, (b) depAu/GCE, (c) H3/depAu-GCE, (d) MCH/H3/depAu/GCE, (e) H2/MCH/H3/depAu/GCE.

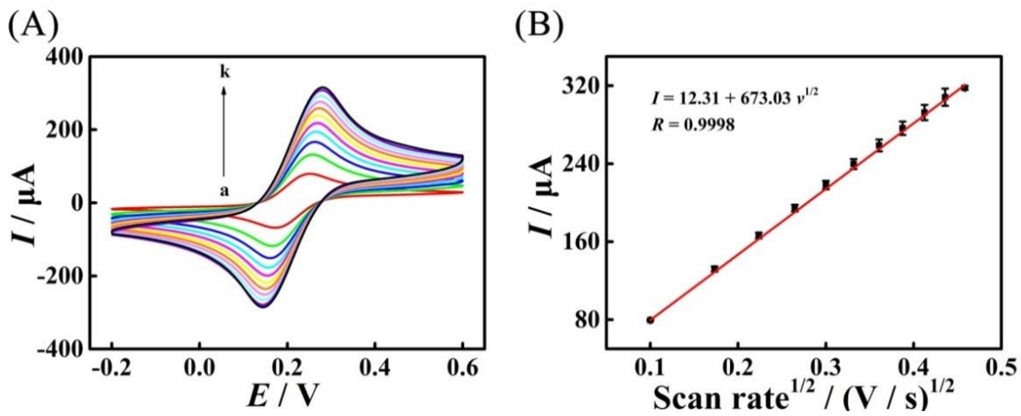

Figure S9. Electroactive area of the modified electrode. (A) CVs of depAu/GCE in 5.0 mM $Fe(CN)_6^{3-/4-}$ at different scan rates (a-k: 10, 30, 50, 70, 90, 110, 130, 150, 170, 190, and 210 mV/s); (B) Calibration curve for Ip *vs* $\nu^{1/2}$, n=3. Error bar, mean ± SD.

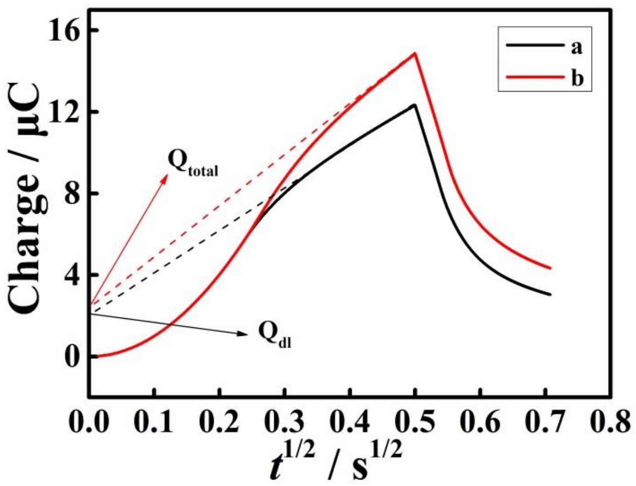

Figure S10. Density of DNA on the modified electrode. Chronocoulometric curves of depAu/GCE modified with H3 (2 μM) (a) in 10 mM Tris-HCl (pH 7.4) and (b) in 10 mM Tris-HCl (pH 7.4) containing 50 μM RuHex.

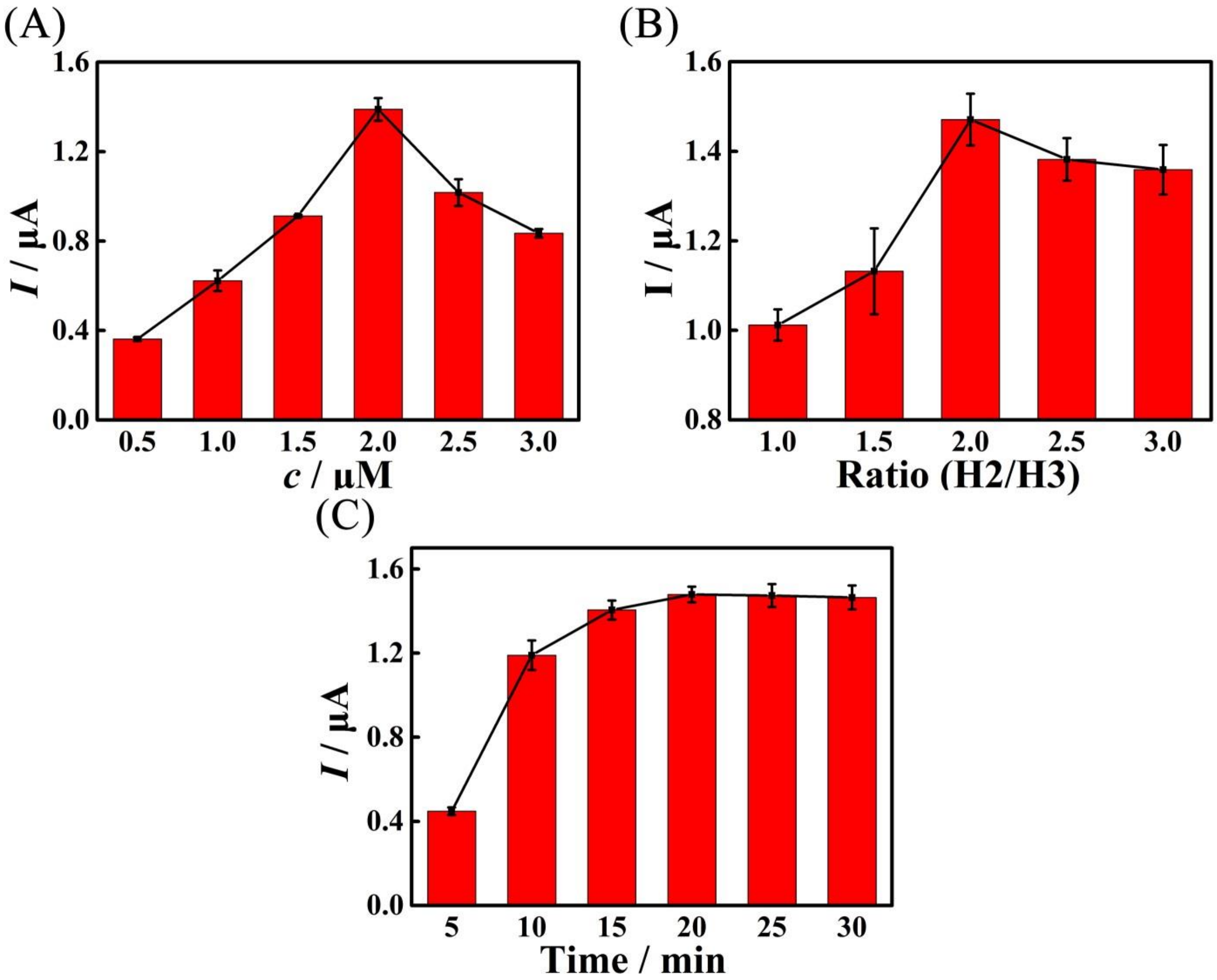

Figure S11. Optimization of Experimental Conditions. Effect on the current response of the proposed biosensor with different experimental parameters: (A) the immobilization concentration of the H3 on the electrode surface (0.5 μM, 1.0 μM, 1.5 μM, 2.0 μM, 2.5 μM, 3.0 μM, with H1 and H2 concentration of 1 μM and miRNA-21 concentration of 1 nM and reaction temperature of 25 ^{o}C, Tris-HCl 7.4), n=3. Error bar, mean ± SD; (B) the ratio of H2 and H3 in DCHA (1.0, 1.5, 2.0, 2.5, 3.0, with H1 and miRNA-21 concentration of 2 μM and 1 nM respectively and reaction temperature of 25 ^{o}C), n=3. Error bar, mean ± SD; (C) the reaction time of DCHA (5 min, 10 min, 15 min, 20 min, 25 min, 30 min), n=3. Error bar, mean ± SD.

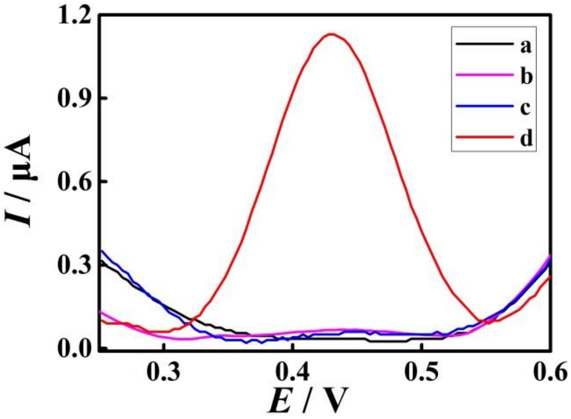

Figure S12. Feasibility of the biosensor in miRNA detection. SWV response of the prepared biosensing platform: (a) with blank sample (without target miRNA, H1, or H2); (b) with target and H2; (c) with H1 and H2; (d) with target miRNA, H1, and H2.

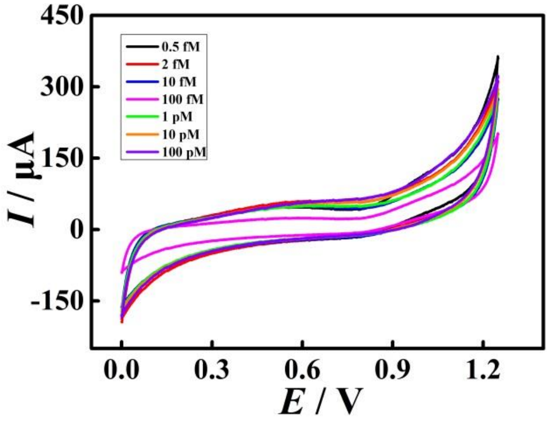

Figure S13. Electrochemical characterization of the prepared ECL biosensor. Typical CV responses of the ECL biosensor under different concentration of miRNA-21 from 0.5 fM to 100 pM.

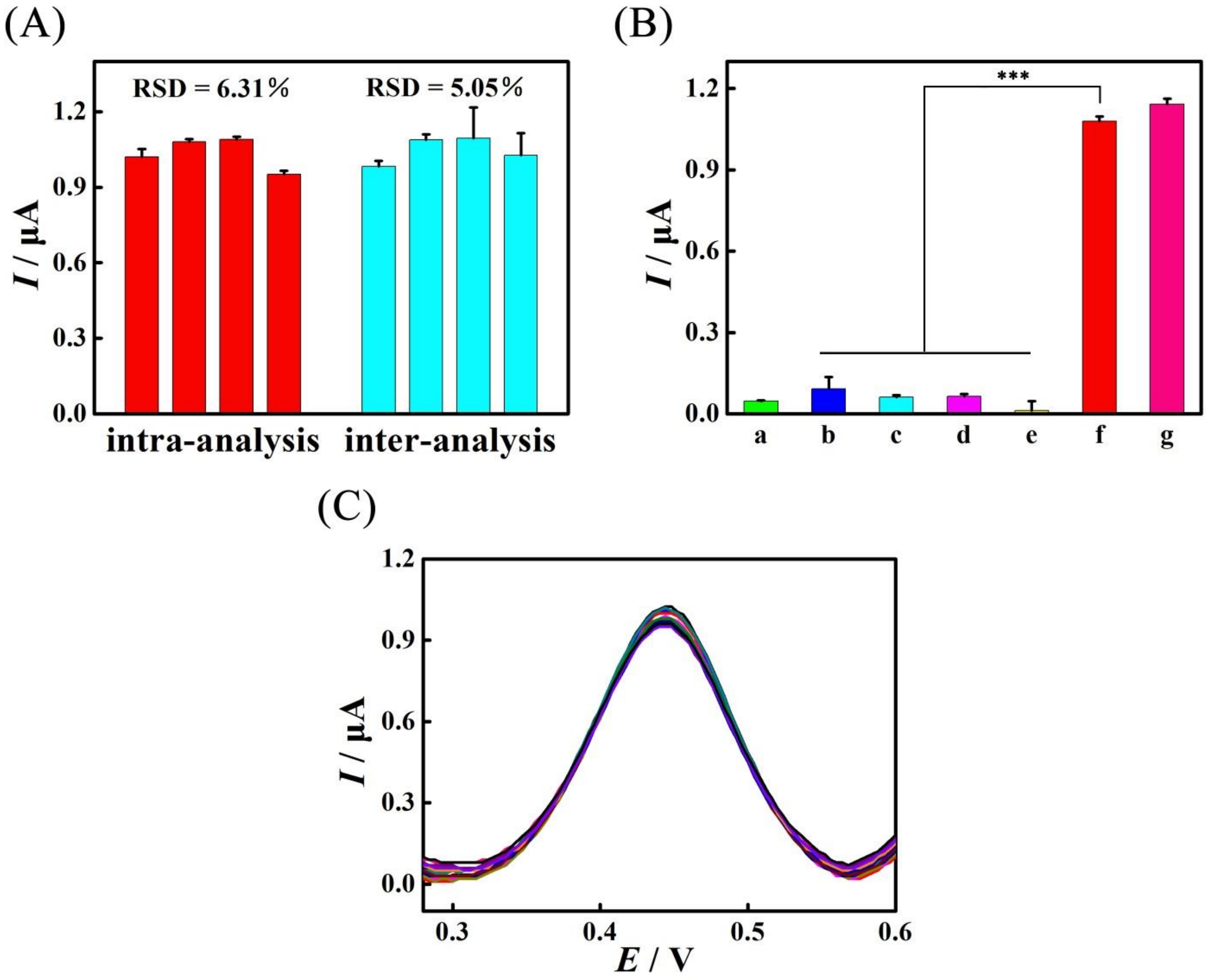

Figure S14. Evaluation of the biosensor performance. (A) The reproducibility (10 pM miRNA-21) of biosensensing platform, n=3. Error bar, mean ± SD. (B) the selectivity of the electrochemical biosensor: (a) blank sample, (b) miRNA-126 (1 nM), (c) miRNA-141 (1 nM), (d) miRNA-155 (1 nM), (e) miRNA-182-5P (1 nM), (f) miRNA-21 (10 pM), and (g) mixed sample, n=3. Error bar, mean ± SD. *P* values are calculated using student T test. **P* < 0.05, ***P* < 0.01, ****P* < 0.001. (C) the stability of the elaborated biosensor (10 pM miRNA-21), n=15.

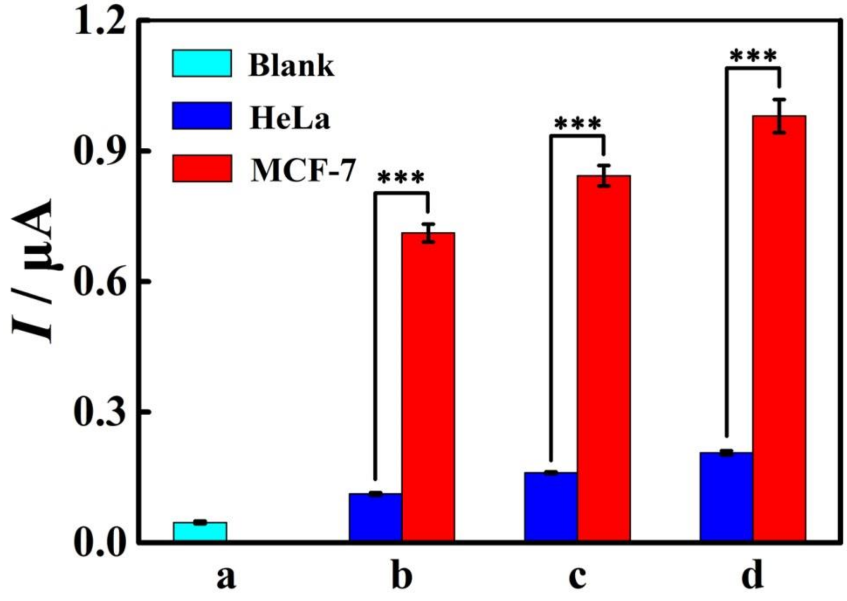

Figure S15. Application of the developed biosensor in cancer cells. Data analysis of the proposed biosensor from different cancer-cell lysates: (a) Blank sample, (b) HeLa (100 cells) and MCF-7 (100 cells), (c) HeLa (1000 cells) and MCF-7 (1000 cells), and (d) HeLa (10000 cells) and MCF-7 (10000 cells) (the cell lysates incubated on the electrode were obtained from such cancer cells with corresponding number, cells were at the same passage number), n=3. Error bar, mean ± SD. *P* values are calculated using student T test. $^{*}P < 0.05$, $^{**}P < 0.01$, $^{***}P < 0.001$.

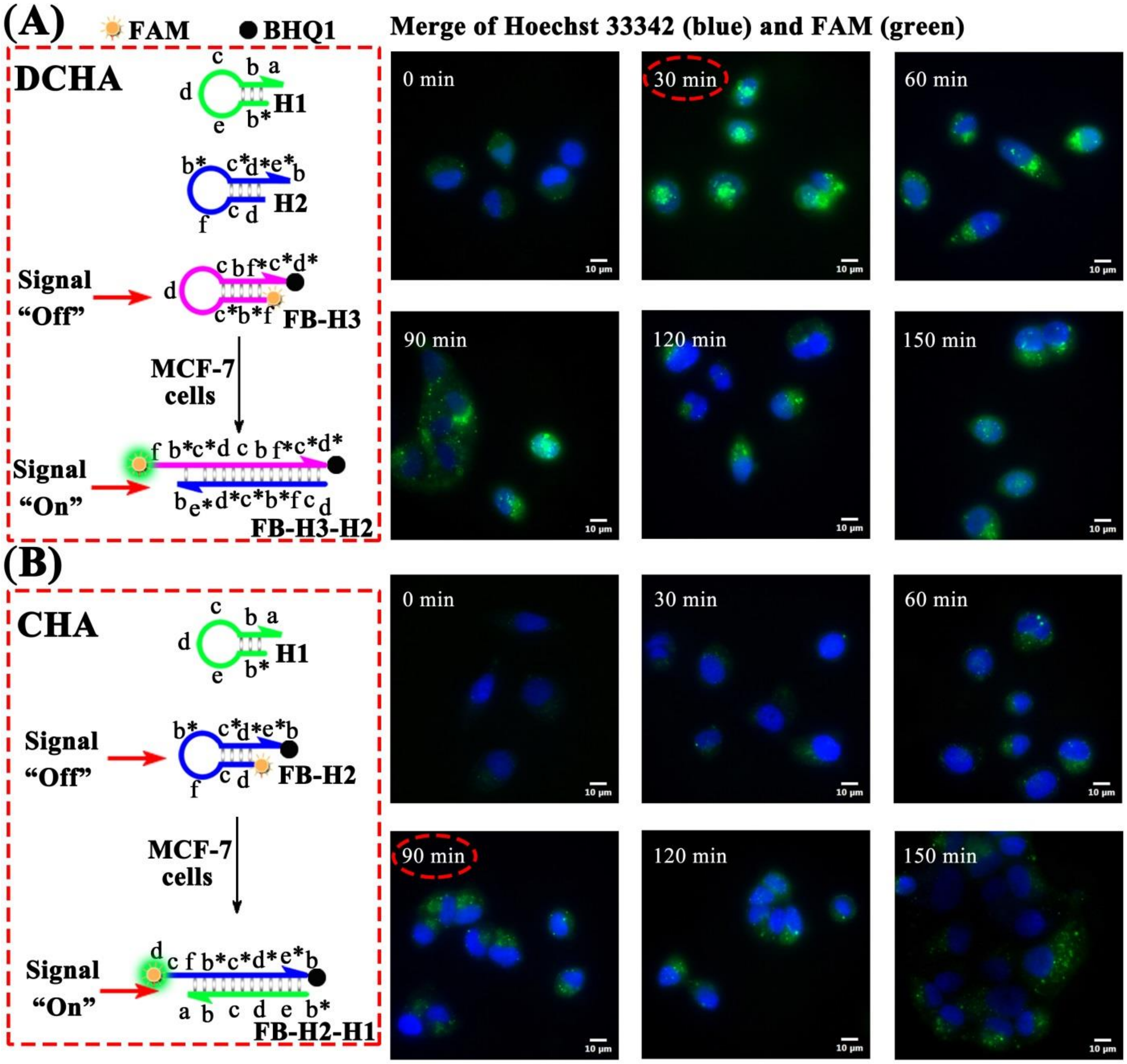

Figure S16. The total internal reflection fluorescence microscope (TIRFM) imaging of miRNAs in living MCF-7 cells. Fluorescence response of (A) DCHA and (B) CHA with different reaction time (0 min, 30 min, 60 min, 90 min, 120 min, 150 min) after the transfection process (concentrations of all the hairpin DNAs were 1 μM, Hoechst were for nuclear staining, cells were at the same passage number).

2.2 Supplemental Tables

Table S1. Synthetic Oligonucleotide Sequences

sequence	from 5' to 3'
H1	ATCAGA(b*) CCC(e) TCAAC(d) ATCAG(c) TCTGAT(b) AAGCTA(a)
H2	Fc-C TCAAC(d) ATCA(c) CATACTT(f) ATCAGA(b*) CTGAT(c*) GTTGA(d*) GGG(e*) TCTGAT(b)
H3	CATACTT(f) ATCAGA(b*) CTGAT(c*) C TCAAC(d) ATCAG(c) TCTGAT(b) AAGTATG(f*) TGAT(c*) GTTGA(d*) G-$(CH_2)_6$-SH
H2'	C TCAAC(d) ATCA(c) CATACTT(f) ATCAGA(b*) CTGAT(c*) GTTGA(d*) GGG(e*) TCTGAT(b)
H3'	CATACTT(f) ATCAGA(b*) CTGAT(c*) C TCAAC(d) ATCAG(c) TCTGAT(b) AAGTATG(f*) TGAT(c*) GTTGA(d*) G
N-H2	NH_2- (CH2)6-C TCAAC(d) ATCA(c) CATACTT(f) ATCAGA(b*) CTGAT(c*) GTTGA(d*) GGG(e*) TCTGAT(b)
DNA-21	TAGCTT(a) ATCAGA(b) CTGAT(c) GTTGA(d)
F-DNA-21	6-FAM- TAGCTT(a) ATCAGA(b) CTGAT(c) GTTGA(d)
B-H1	ATCAGA(b*) CCC(e) TCAAC(d) ATCAG(c) TCTGAT(b) AAGCTA(a)-BHQ1
F-H1	6-FAM- ATCAGA(b*) CCC(e) TCAAC(d) ATCAG(c) TCTGAT(b) AAGCTA(a)
B-H2	C TCAAC(d) ATCA(c) CATACTT(f) ATCAGA(b*) CTGAT(c*) GTTGA(d*) GGG(e*) TCTGAT(b)-BHQ1
F-H2	6-FAM- C TCAAC(d) ATCA(c) CATACTT(f) ATCAGA(b*) CTGAT(c*) GTTGA(d*) GGG(e*) TCTGAT(b)

B-H3	CATACTT(f) ATCAGA(b*) CTGAT(c*) C TCAAC(d) ATCAG(c) TCTGAT(b) AAGTATG(f*) TGAT(c*) GTTGA(d*) G -BHQ1
FB-H2	6-FAM- C TCAAC(d) ATCA(c) CATACTT(f) ATCAGA(b*) CTGAT(c*) GTTGA(d*) GGG(e*) TCTGAT(b)-BHQ1
FB-H3	6-FAM- CATACTT(f) ATCAGA(b*) CTGAT(c*) C TCAAC(d) ATCAG(c) TCTGAT(b) AAGTATG(f*) TGAT(c*) GTTGA(d*) G -BHQ1
miRNA-21	UAGCUU(a) AUCAGA(b) CUGAU(c) GUUGA(d)
miRNA-126	CAUUAUUACUUUUGGUAC
miRNA-141	UAACACUGUCUGGUAAAGAUGG
miRNA-155	UUAAUGCUAAUCGUG AUAGGGGU
miRNA-182-5p	UUU GGC AAU GGU AGA ACU CAC ACU

Table S2. Experimental Conversion Efficiency N of DCHA and CHA with Different Concentration of Initiator by Fluorescence (F) and Electrochemistry (E) respectively (T = 25 °C, $c_{H1} = c_{H2} = c_{H3}$ = 1 μM (F); $c_{H1} = c_{H3}$ = 2 μM and c_{H2} = 4 μM (E), n=3)

Initiator concentration /M	CHA (F)	DCHA (F)	DCHA (E)
6.0×10^{-16}	-	-	454498247.1
2.0×10^{-15}	-	-	204562181.2
1.0×10^{-14}	-	30493826	59149412.42
1.0×10^{-13}	-	4138480.4	8524062.675
1.0×10^{-12}	290976	522757.82	1113318.411
1.0×10^{-11}	39078.3	63166.76	137423.0554
1.0×10^{-10}	4905.91	7405.7738	16351.42697
1.0×10^{-9}	590.398	849.48716	1896.054841
1.0×10^{-8}	69.0205	-	-
1.0×10^{-7}	7.90013	-	-

Table S3. Limit of Detection Calculation for the miRNA Biosensing (n=10)

Biosensors	x_B	s_B	I_L (F_L)	Detection Limit
Fluorescence (CHA) / a.u.	2393.00	66.65	2193.05	0.36 pM
Fluorescence (DCHA) / a.u.	2329.00	80.97	2086.10	6.21 fM
Electrochemical (DCHA) / μA	0.14	2.34×10^{-8}	0.21	0.34 fM
ECL (DCHA) / a.u.	1309.70	137.72	1722.86	0.19 fM

References

[1] D. Zhang, E. Winfree, *J. Am. Chem. Soc.* **2009**, *131*, 17303-17314.

[2] T. Song, S. Xiao, D. Yao, F. Huang, M. Hu, H. Liang, *Adv. Mater*. **2014**, *26*, 6181-6185.

[3] K. Umezawa, M. Yoshida, M. Kamiya, T. Yamasoba, Y. Urano, *Nat. Chem.* **2016**, *9*, 279-286.

[4] C. A. Figg, P. H. Winegar, O. G. Hayes, C. A. Mirkin, *J. Am. Chem. Soc*. **2020**, *142*, 8596-8601.

[5] F. Wang, C. Ye, S. Mo, H. Luo, J. Chen, Y. Shi, N. Li, *Anal. Bional. Chem.* **2019**, *411*, 3059-3068.

[6] J. Singh, A. Roychoudhury, M. Srivastava, P. R. Solanki, D. W. Lee, S. H. Lee, B. D. Malhotra, *Nanoscale* **2014**, *6*, 1195-1208.

[7] S. Kundu, H. Liang, *Adv. Mater* **2008**, *20*, 826-831.

[8] J. Zhang, S. Song, L. Wang, D. Pan, C. Fan, *Nat. Protoc.* **2007**, *2*, 2888-2895.

[9] A. Radi, J. L. Acero Sánchez, E. Baldrich, C. K. O'Sullivan, *J. Am. Chem. Soc.* **2006**, *128*, 117-124.

[10] Z. Wang, J. Han, Y. Cui, X. Zhou, K. Fan, *Biochem. Biophys. Res. Commun.* **2013**, *439*, 384-389.

[11] S. Li, L. Xu, W. Ma, X. Wu, M. Sun, H. Kuang, L. Wang, N. A. Kotov, C. Xu, *J. Am. Chem. Soc.* **2016**, 138, 306-312.

[12] M. V. Iorio, M. Ferracin, C. Liu, A. Veronese, R. Spizzo, S. Sabbioni, E. Magri, M. Pedriali, M. Fabbri, M. Campiglio, S. M´enard, J. P. Palazzo, A. Rosenberg, P. Musiani, S. Volinia, I. Nenci, G. A. Calin, P. Querzoli, M. Negrini, C. M. Croce, *Cancer Res*. 2005, 65, 7065-7070.

[13] F. Yang, Y. R. Cheng, Y. Cao, H. F. Dong, H. T. Lu, K. Zhang, X. D. Meng, C. H. Liu, X. J. Zhang, *Chem. Sci.* **2019**, 10, 1709-1715.
